# Comparison between the UV and X-ray Photosensitivities of Hybrid TiO_2_-SiO_2_ Thin Layers

**DOI:** 10.3390/ma13173730

**Published:** 2020-08-24

**Authors:** Maxime Royon, Francis Vocanson, Damien Jamon, Emmanuel Marin, Adriana Morana, Aziz Boukenter, Sylvain Girard, Youcef Ouerdane, François Royer, Yves Jourlin

**Affiliations:** Univ Lyon, Laboratoire H. Curien, UJM-CNRS-IOGS, 18 rue du Pr. 42000 Benoît Lauras, Saint-Etienne, France; francis.vocanson@univ-st-etienne.fr (F.V.); damien.jamon@univ-st-etienne.fr (D.J.); emmanuel.marin@univ-st-etienne.fr (E.M.); adriana.morana@univ-st-etienne.fr (A.M.); aziz.boukenter@univ-st-etienne.fr (A.B.); sylvain.girard@univ-st-etienne.fr (S.G.); ouerdane@univ-st-etienne.fr (Y.O.); francois.royer@univ-st-etienne.fr (F.R.); yves.jourlin@univ-st-etienne.fr (Y.J.)

**Keywords:** sol–gel materials, X-ray photolithography, FTIR spectroscopy, photopolymerization

## Abstract

The photo-induced effects on sol–gel-based organo TiO_2_-SiO_2_ thin layers deposited by the dip-coating technique have been investigated using two very different light sources: A light-emitting diode (LED) emitting in the UV (at 365 nm, 3.4 eV) and an X-ray tube producing 40 keV mean-energy photons. The impact of adding a photo-initiator (2,2-dimethoxy-2-phenylacetophenone-DMPA) on the sol–gel photosensitivity is characterized namely in terms of the photo-induced refractive index measured through M-line spectroscopy. Results show that both silica-titania sol–gel films with or without the photo-initiator are photosensitive to both photon sources. The induced refractive index values reveal several features where slightly higher refractive indexes are obtained for the sol–gel containing the photo-initiator. UV and X-ray-induced polymerization degrees are discussed using Fourier-transform infrared (FTIR) spectroscopy where the densification of hybrid TiO_2_-SiO_2_ layers is related to the consumption of the CH=C groups and to the decomposition of Si-OH and Si-O-CH_3_ bonds. X-rays are more efficient at densifying the TiO_2_-SiO_2_ inorganic and organic network with respect to the UV photons. Hard X-ray photolithography, where no cracks or damages are observed after intense exposition, can be a promising technique to design submicronic-structure patterns on TiO_2_-SiO_2_ thin layers for the building of optical sensors.

## 1. Introduction

The sol–gel chemistry process, based on hydrolysis and condensation reactions [1], is an elegant technique used for the low-temperature synthesis of optical materials. This soft chemistry route allows the fabrication of various optical elements in large applicative fields including the fabrication of waveguides with a very high transparency in the telecommunication ranges [2,3,4], and diffraction gratings [5,6] whose applications can be observed in solar applications [7]. It is even possible to create Bragg gratings using the sol–gel approach through nanoimprinting processes [8], revealing the potentiality to use this structure as strain and temperature sensors as it can be observed with bulk silica glass or optical fibers [9,10]. Typically, metal alkoxides precursors (Ti, Zr, Si….) are used to form an inorganic metal oxides matrix (TiO_2_, ZrO_2_, SiO_2_…) or more hybrid structures. By varying the chemical conditions and the molar ratio, the morphology of the induced materials is drastically affected. It is thus possible to tailor their mechanical and optical properties namely in terms of absorption and refractive index but also confer additional functionalities to the films by incorporation of molecules or network modifiers [11]. The literature has largely described the behavior and the UV photosensitivity of sol–gel materials by the addition of a photo-initiator during the preparation of the mixture [12,13]. More specifically, the interaction of light with the material can be divided into two main classes depending on the insolated photoresist. The first class corresponds to negative photoresists where the insolated portion is polymerized under light exposure to form a densified and insoluble pattern. The second class acts as positive photoresists. For this latter, the portion exposed to the radiation is removed during the development process. Even if the UV photolithography is still widely used, the existence of unconventional techniques (electron beam and X-ray photolithography) opens the way to very-high-resolution structures at the nanometer scale and offers the possibility to functionalize materials in extended and new ranges of fields. For example, very small and precise features in the 5 nm range were obtained with PMMA and inorganic metal halides [14]. X-ray photolithography was also studied on both positive [15,16] and negative photoresists [17,18]. In [15], results showed that an organic–inorganic sol–gel was photosensitive to soft X-ray synchrotron radiation with photon energies ranging from 1 to 4 keV and that the obtained patterns had lateral dimensions close to 100 nm [16]. Another study performed on negative silica sol–gel films (methyltriethoxysilane-tetraethoxysilane) exposed to harder X-rays (2.5–12 keV) revealed an important densification of the layers [18]. These studies clearly show the benefits of such kind of radiations and open the way to the exploitation of sol–gel films to create sub-micrometric structures in the field of miniaturized sensors where the refractive index remains a crucial parameter. However, to our knowledge, there are no studies regarding the effect of X-ray irradiation performed on TiO_2_-SiO_2_ thin films. To this aim, we have performed a study on two hybrid TiO_2_-SiO_2_ layers. The mixture is strictly the same; however, to highlight the possible benefit of the photosensitizer on the optical properties after X-ray exposure, the first one contains a photo-initiator at a fixed concentration, whereas the second is photo-initiator-free. Both sol–gel layers were exposed to hard X-ray photons (40 keV) and UV photons (365 nm–3.4 eV) in order to check the induced photosensitivity. Further investigations on these films have been carried out by M-line spectroscopy to have access to the induced refractive index, and Fourier-transform infrared (FTIR) spectroscopy, revealing the optical and structural modifications caused by the UV or X-ray exposure.

## 2. Materials and Methods

**Figure 1:**
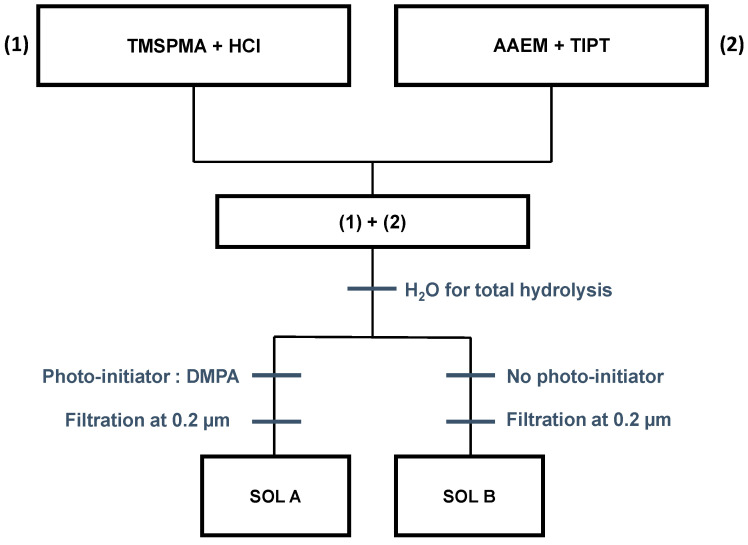
Preparation of TiO_2_-SiO_2_ sol–gel. Sol A represents the sol–gel containing the UV photo-initiator (2,2-dimethoxy-2-phenylacetophenone). Sol B was prepared following the same procedure but without the photo-initiator.

For all the experiments, we have used a homemade and hybrid sol–gel composed of TiO_2_-SiO_2_ whose preparation is shown in Figure 1. This sol–gel was developed and optimized for the photolithography tools we have in the lab, namely in terms of wavelength and power. All the chemical reagents were provided by Sigma-Aldrich (Saint-Quentin Fallavier, France) and used without purification. The sol–gel was created using 3-(trimethoxysilyl)propyl methacrylate (TMSPMA) and titanium (IV) isopropoxide (TIPT) as silicate and titanium precursors, respectively. A partial hydrolysis and condensation of 3-(trimethoxysilyl)propyl methacrylate was achieved using acidified water (HCl). In addition, 2-(methacryloyloxy)ethyl acetoacetate (AAEM), working as the chelated agent, and titanium (IV) isopropoxide were added in (1). The molar ratios of precursors Si:Ti:AAEM are 10:10:5.5. After a stirring period of 1 h, a total hydrolysis is performed through the addition of water. A 2,2-dimethoxy-2-phenylacetophenone (DMPA) photo-initiator in the form of 0.7 wt.% was added in the dark to initiate a UV photopolymerization as it is extensively described in the literature [12,13,19]. DMPA was chosen as it has been identified as an efficient and stable molecule with a high reactivity with this kind of formulation. The mixture is then filtered at 0.2 µm. After an outgassing under nitrogen atmosphere, the solution was aged for 48 h before use. This procedure denotes the “sol A” as shown in Figure 1. In order to investigate the impact of the photo-initiator, a solution named “sol B” was prepared following the same procedure but without the photo-initiator.

**Figure 2:**
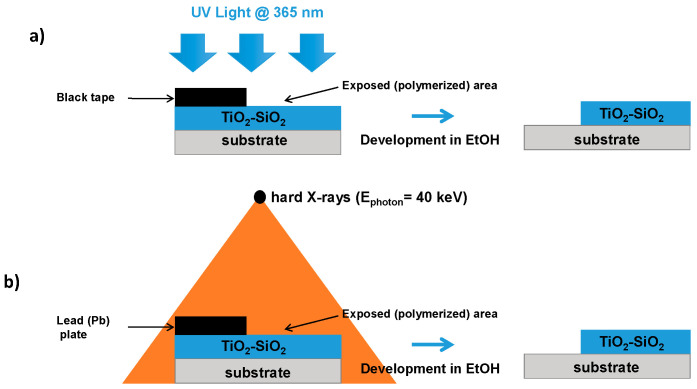
Photosensitivity test for TiO_2_-SiO_2_ using: (**a**) The UV lamp (UWAVE) emitting at 365 nm, (**b**) the MoperiX facility delivering hard X-ray photons (40 keV). In both cases, the development is made in ethanol solution.

The TiO_2_-SiO_2_ layers were deposited on two different substrates (Borofloat 33 and silicium) that were cleaned according to three steps of soaking in acetone, ethanol, and deionized water before drying under nitrogen. The sols were all deposited in a clean room at a fixed temperature and hygrometry using the dip-coating approach at a translation speed of 7 cm/min, resulting in a film thickness of 4.9 µm. The deposited films were then baked at 60 °C for 5 min in order to evaporate the solvent and to make a pre-densification of the TiO_2_-SiO_2_ layers. For the refractive index measurement, the films were deposited on a Borofloat 33 substrate provided by Neyco (Vanves, France). Indeed, the M-line spectroscopy used for the refractive index estimation is based on the propagated mode inside the layer. It is thus essential to have an important refractive index change between the layer and the substrate to ensure adequate guiding properties. For example, the refractive indexes are 1.58 and 1.47 at 633 nm for the UV-insolated layer and the Borofloat 33 glass, respectively. The films were also characterized by Fourier-transform infrared (FTIR) spectroscopy. To this aim, the films were deposited on a Si substrate, ideal for use in the far-IR region. To investigate the effect of different light sources on the photosensitivity of our homemade films, two sources operating in different wavelength ranges were used. The first one was a UV source (UWAVE) emitting at a 365 nm wavelength composed by an LED matrix resulting in a 10 cm square light source (Figure 2a). For this latter, it is possible to control the power and the exposure time. We also used the MoperiX facility from the Laboratoire Hubert Curien (Figure 2b). The X-ray photons were produced by the Bremsstrahlung effect, and we have chosen a 100 kV voltage between the anode and the cathode, resulting in the creation of hard X-rays with a mean photon energy of about 40 keV (0.3 Å). In this case, the distance between the source and the layers determines the dose rate (Gy/s) and, by extension, the accumulated dose (1 Gy = 1 J/kg). The dose rate can strongly impact the optical response of the material submitted to X-rays. For instance, such dose rate dependence has been observed regarding pure-silica core fibers and germanosilicate fibers [20]. In order to avoid this behavior, we have fixed the dose rate at 14 Gy/s for all the accumulated doses presented in this work. It should be noted that the measured dose rate is constant over the entire layers, revealing that the films are homogeneously irradiated. Moreover, a previous study regarding the hard X-ray (40 keV) exposure on bulk silica glass showed that they are mainly stopped in the first half mm inside the sample [21]. With the thicknesses of the sol–gel layers being around 5 µm, a high penetration depth is possible, revealing the possibility to obtain high-aspect-ratio structures. The TiO_2_-SiO_2_ layer reacts as a negative photoresist insofar as the zones where the layer has not been exposed and polymerized with the light source are dissoluted during the development in an alcoholic solution such as ethanol. Regarding the UV exposure, a black tape is used to prevent the UV-induced polymerization process on a small area of the layer, as shown in Figure 2a. It is thus possible to determine the minimum fluence needed for a full polymerization of the layer. However, concerning the X-ray exposure, a lead plate should be used to cover the layer as illustrated in Figure 2b. The development is realized in an EtOH bath where the polymerized area is not dissoluted, as represented in Figure 2b.

**Figure 3:**
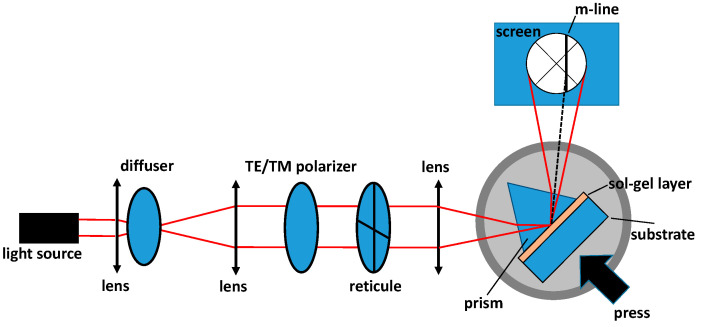
M-line spectroscopy used for the effective refractive index of the TiO_2_-SiO_2_ sol–gel layers. A 633 nm light source is used.

As previously mentioned, one of the objectives of the photolithography technique is the control of the refractive index evolution fostering better performances of the photonic devices. In order to investigate the refractive index as a function of the light sources used for the photolithography and the impact of the photo-initiator, we have used the M-line spectroscopy widely detailed in the literature [22], based on LASF35 isosceles prism coupling and allowing the determination of the propagated mode effective refractive index inside the layer. The setup used for the characterization is shown in Figure 3. To this purpose, a 633 nm wavelength source is collimated and focused onto the base of a prism located on a rotating plate for precise angle measurements. A polarizer is placed on the optical path allowing adjustment of the TE (transverse electric) or TM (transverse magnetic) mode. The reflected visible light is then observed on a camera where the guided modes launched into the TiO_2_-SiO_2_ films manifest themselves as black lines. The rotation of the goniometer, which is the location of the prism, the thin layer, and the press, allows us to precisely align the reticule with a black line and to have access to the corresponding angle. Their observation allows the determination of the effective refractive index with a precision of 10^−3^. For a given sample, this quantity is an average value of two measurements obtained by removing and replacing the sol–gel layers between the press and the prism. In addition to the M-line spectroscopy, we have characterized the thin layers using a Nicolet iS20 FTIR spectrometer (Thermo Scientific, Waltham, Massachusetts, USA) in attenuated total reflectance (ATR) mode with a 0.482 cm^−1^ resolution.

## 3. Results

### 3.1. Photosensitivity Tests

**Figure 4:**
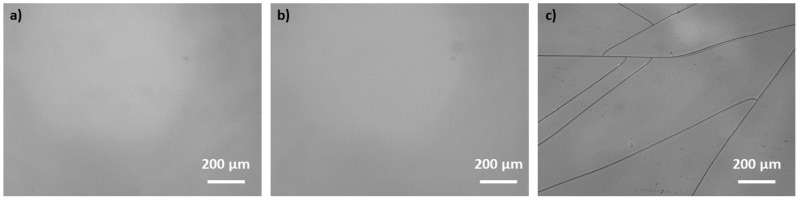
Microscope images (X10) of silica-titania sol–gel layers obtained in reflexion mode for: (**a**) sol–gel irradiated with UV photons (180 J/cm^2^), (**b**) sol–gel irradiated with 40 keV hard X-rays at a 100 kGy accumulated dose, and (**c**) baked sol–gel at 90 °C during 2 h (no light exposure).

In order to check the photosensitivity of our sol–gel layers, we have performed several X-ray irradiations on sol A (photo-initiator DMPA) and B (photo-initiator free), as described in Table 1, with accumulated doses ranging from 0.5 to 100 kGy. Regarding the sol A response to X-rays, three regimes can be discerned. The first regime occurs when using low accumulated doses ranging from 0.5 to 10 kGy where sol–gel layers do not seem to be sufficiently insolated: The unexposed and exposed areas are both dissolved during the development process. Beyond this value, a polymerization is observed for a 15 kGy total accumulated dose. However, for this latter, the polymerization remains partial insofar as an important area of the exposed sol–gel layer is also removed, meaning that we have reached a threshold transition zone. The fact that the exposed regions are removed after development in EtOH solution is mainly attributed to the minor cross-linking effect for this range of accumulated doses. For higher ones (25, 50, and 100 kGy), a full polymerization is induced. The same experiment was repeated using sol B, referring to the sol–gel without the photo-initiator. Interestingly, it should be noted that the DMPA photo-initiator seems to not influence the photosensitivity of the layers, the same behavior as for sol A being obtained: a full polymerization is also achieved at 25 kGy. The behavior of hybrid TiO_2_-SiO_2_ surface layers submitted to X-rays is not well known. As observed through the microscope image given in Figure 4b, no surface damage or cracking was present after the X-ray-induced densification, even at the maximum accumulated dose (100 kGy) as it was previously reported in [18] on hybrid silica layers. Moreover, no damages were induced for the UV-cured sol–gel (Figure 4a). Contrary to UV or X-ray exposure, a high thermal treatment can have a strong negative impact on the TiO_2_-SiO_2_ surface layers. In order to highlight this behavior, we have applied a bake process after the deposition (no light exposure) at 90 °C during 2 h where cracks and surface damages are observed mainly due to tensile stress, as demonstrated in Figure 4c. This comparison between light exposure and thermal treatment clearly evidences that organo TiO_2_-SiO_2_ layers are less affected in terms of damages by hard X-ray photons than by a thermal treatment and that they can act as good candidates for X-ray lithography even at very high 40 keV photon energies.

The two different sol–gels were also characterized through UV insolation (Figure 2a). The results show that they are both polymerized with a minimum threshold fluence of 90 J/cm^2^. Below this value, the layers are totally dissoluted during the development process and follow the same trend observed with X-ray exposure: the amount of the photo-initiator does not seem to affect the behavior of the materials in terms of photosensitivity. This can be explained by the DMPA concentration of 0.7 wt.% used for this experiment. Indeed, with a higher concentration ranging from 3 to 5 wt.% as it can be observed in the literature [12], the photodegradation of the exposed films can be accelerated, and a difference in the polymerization threshold between DMPA and DMPA-free solutions can be observed.

### 3.2. Refractive Indexes Measurements

**Figure 5:**
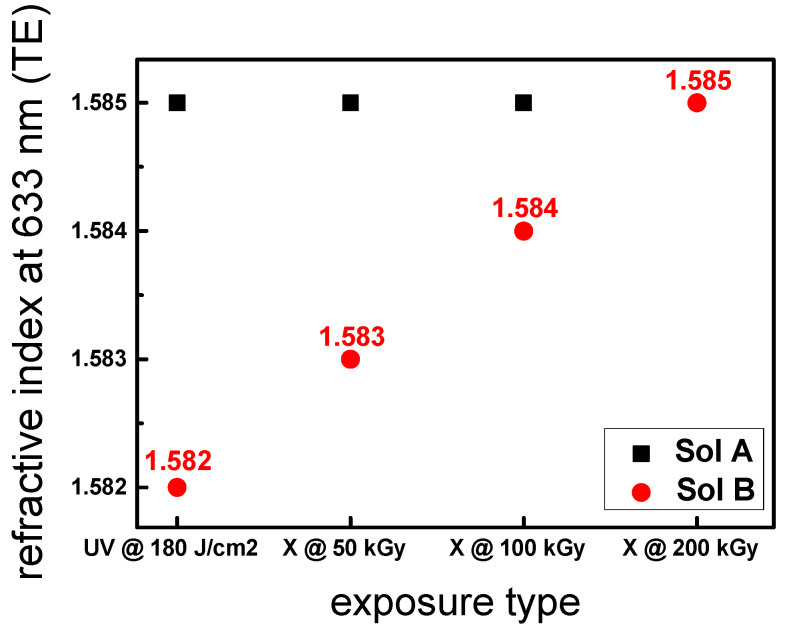
Effective refractive index of the layers (sol A and B) at 633 nm in transverse electric (TE) mode for different exposures: UV (180 J/cm^2^) and X-rays (50, 100, and 200 kGy). The films were deposited on a Borofloat 33 substrate (n = 1.47 at 633 nm) to ensure an important refractive index change between the layer and the substrate. The precision of the refractive index measurements is 10^−3^ insofar as a high number of guided modes are observed.

In addition to the photosensitivity tests, the different layers were characterized in terms of refractive index at 633 nm for the TE mode in order to have access to a potential effect of the photo-initiator. Figure 5 shows the induced refractive index for sol A and B using M-line spectroscopy. Through this graph, we can clearly see the effect of the photo-initiator. Concerning sol A (black square), the refractive index is constant (1.585) whatever the source (UV or X-rays) used for the polymerization. It is worth noting that for the layers irradiated in the X-ray regime, whatever the dose, the effective refractive index is stable even at higher accumulated doses (100 kGy). In the absence of the photo-initiator (red dot), the refractive index of the layer is 1.582 using UV light at 180 J/cm^2^. This value is lower compared that of sol A (1.585), highlighting the effect of the photo-initiator. The same trend can be observed for the layers irradiated with X-rays where the refractive indexes of sol B are globally lower.

Depending on the accumulated dose, the refractive index slightly increases with a slope rate of 2.10^−5^ kGy^−1^. In order to validate this behavior, we have also irradiated sol B with a 200 kGy total accumulated dose where no cracks are observed after exposure. This result confirms a good agreement with the previous data (50 and 100 kGy) where a higher effective refractive index of 1.585 is obtained corresponding to the maximum value achievable at 633 nm.

### 3.3. FTIR Spectroscopy

**Figure 6:**
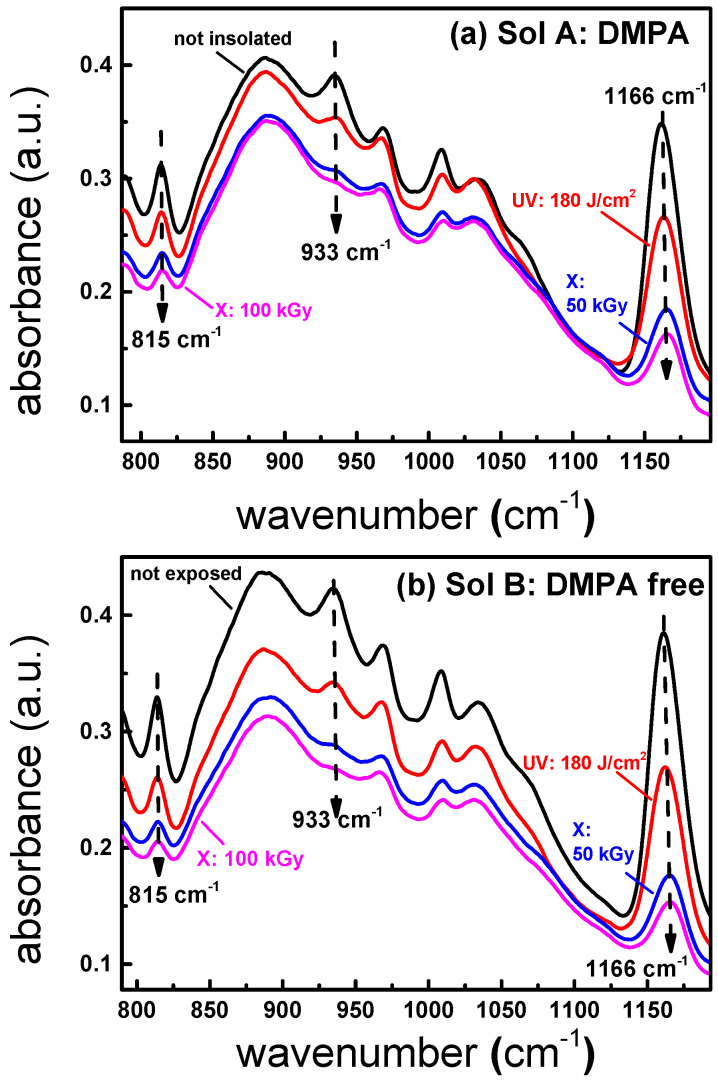
FTIR spectra of sol A with the 2,2-dimethoxy-2-phenylacetophenone (DMPA) photo-initiator (**a**) and sol B without DMPA (**b**). Both cases represent the non-exposed films (black), the UV-exposed films at 180 J/cm^2^ (red), and the X-ray-exposed films at 50 kGy (blue) and 100 kGy (magenta).

The effect of UV and X-ray radiations has also been evaluated by FTIR spectroscopy in order to investigate the chemical and structural changes in the hybrid thin films. As previously mentioned, the films should be deposited on Si substrates insofar as it is transparent in the far-IR region. To investigate the potential impact of hard X-rays on Si wafer, we have performed Raman spectroscopy measurements on different Si substrates submitted to accumulated doses ranging from 50 to 100 kGy. The results show that the band peaking at roughly 520 cm^−1^ corresponding to the vibrational mode of Si is not affected in terms of wavenumber and full-width at half-maximum compared to the pristine sample. This result clearly reveals that there are no structural changes on Si substrates after X-ray exposure in this accumulated dose range, as it was already shown in [23], where Si nanocrystals are not altered up to 300 kGy. FTIR spectroscopy is a powerful technique in order to monitor the light-induced structural changes and polymerization in the inorganic network of various thin films. The literature has extensively studied the behavior of TiO_2_-SiO_2_ layers through this technique [24,25,26] where inorganic and organic network modifications related to photopolymerization are mainly observed in the 800–1300 cm^−1^ range. We report in Figure 6a,b the FTIR spectra obtained for sol A and B, respectively, submitted to different exposures: non-exposed films (black line), UV-cured films at 180 J/cm^2^ (red line), and hard X-ray-exposed films at 50 kGy (blue line) and 100 kGy (magenta line). After UV or X-ray exposure, the sol A spectra (Figure 6a) show several features through three bands peaking at 815, 933, and 1166 cm^−1^. The first band located at roughly 815 cm^−1^ highlights the photoinduced organic network formation and corresponds to the stretching vibration of CH=C. Under UV treatment, the band intensity decreases compared to the pristine layer, indicating the consumption of the CH=C double bonds and, consequently, a polymerization process. It should be noted that the area under this peak is even lower when the layer is X-ray-irradiated at a 50 and 100 kGy accumulated dose, confirming a more important polymerization rate using this kind of radiation. The decrease in the 815 cm^−1^ band after light exposure was already reported in the literature and used to control the photo-induced polymerization [25]. FTIR measurements can also identify the structural changes that occur in the inorganic network, namely in terms of Si-O-Ti and Si-O-Si bonds. Figure 6a reveals two bands peaking at 1166 and 933 cm^−1^ when the sol–gel layer is not exposed to light. In both cases, we can observe a decrease in these bands with respect to UV or X-ray exposures. The first one, peaking at 1166 cm^−1^, corresponds to the stretching vibration of Si-O-CH_3_. The dramatic decrease in this band with respect to UV or X-ray exposure indicates the formation of Si-O-Ti or Si-O-Si bonds, revealing a higher polymerization. Another interesting feature of Figure 6a is the band located at 933 cm^−1^.This band is attributed to the presence of silanol groups (Si-OH). The decrease in this band with respect to photon exposure denotes a consumption of hydroxyl groups. As this band is very weak upon X-ray exposure, it clearly indicates that X-ray photons are more efficient to decompose Si-OH and Si-O-CH_3_ bonds, leading to a densification of the inorganic network through two main bonds: Si-O-Si and Si-O-Ti. Sol B was also characterized through FTIR spectroscopy as it can be shown in Figure 6b. It is interesting to note that, even without the use of photo-initiator, the sol–gel is still photosensitive to our UV and X-ray treatments following the same behavior as Sol A, namely in terms of photoinduced changes related to the bands peaking at 815, 933, and 1166 cm^−1^.

## 4. Conclusions

In the present work, we have investigated the effect of different light sources (UV and hard X-rays) on the polymerization of two TiO_2_-SiO_2_ sol–gels by comparing the influence of a commercial photo-initiator (2,2-dimethoxy-2-phenylacetophenone) on the film properties. It was observed that the layers are photosensitive with respect to 3.4 eV or 40 keV photons even if the photo-initiator is absent. After X-ray exposure, the films reveal no cracks or damaged surfaces, leading to the possibility of using such sources in order to polymerize sol–gel layers. By comparing the exposed layers in terms of refractive indexes, we have highlighted the effect of the photo-initiator where slightly higher and constant refractive indexes are achieved when DMPA is incorporated in the sol–gel compared to a DMPA-free solution. For this latter, higher refractive index values are obtained when using hard X-ray photons to reach a maximum value of 1.585 at 633 nm. It should be noted that the concentration of the photo-initiator does not seem to affect the behavior of the materials in terms of photosensitivity where the polymerization threshold remains strictly the same with and without the photo-initiator. This behavior can be explained by the DMPA amount of 0.7 wt.% used for this experiment that is too low and that more important concentrations should be investigated in the future to see a potential difference. FTIR measurements evidence the positive impact of X-rays through a stronger densification of the inorganic network induced by the decomposition of Si-OH and Si-O-CH_3_ bonds compared to UV photons. These promising results show that hard X-ray micro- or nano-beams can be considered in the future as an efficient tool in order to microstructure or nanostructure complex patterns on hybrid TiO_2_-SiO_2_ beyond the UV light diffraction limit.

## Figures and Tables

**Table 1:** Effect of X-ray accumulated dose (kGy) on the photopolymerization of sol A (photo-initiator) and B (photo-initiator free).

Accumulated Dose (kGy)	Sol A Polymerization	Sol B Polymerization
0.5	No	No
5	No	No
10	No	No
15	Partial	Partial
25	Full	Full
50	Full	Full
100	Full	Full

## References

[1] Hench L.L., West J.K. (1990). The sol-gel process. Chem. Rev..

[2] Coudray P., Etienne P., Moreau Y., Porque J., Najafi S. (1997). Sol-gel channel waveguide on silicon: Fast direct imprinting and low cost fabrication. Opt. Commun..

[3] Oubaha M., Kribich R.K., Copperwhite R., Etienne P., O’Dwyer K., MacCraith B.D., Moreau Y. (2005). New inorganic sol-gel material with high transparency at 1.55 µm. Opt. Commun..

[4] Versace D., Oubaha M., Copperwhite R., Croutxé-Barghorn C., MacCraith B. (2008). Waveguide fabrication in UV-photocurable sol–gel materials: Influence of the photoinitiating system. Thin Solid Film.

[5] Gâté V., Jourlin Y., Vocanson F., Dellea O., Vercasson G., Reynaud S., Riassetto D., Langlet M. (2013). Sub-micrometric patterns written using a DIL method coupled to a TiO2 photo-resist. Opt. Mater..

[6] Gâté V., Bernaud G., Veillas C., Cazier A., Vocanson F., Jourlin Y., Langlet M. (2013). Fast dynamic interferometric lithography for large submicrometric period diffraction gratings production. Opt. Eng..

[7] Gombert A., Rose K., Heinzel A., Horbelt W., Zanke C., Bläsi B., Wittwer V. (1998). Antireflective submicrometer surface-relief gratings for solar applications. Sol. Energy Mater. Sol. Cells.

[8] Casalboni M., Dominici L., Foglietti V., Michelotti F., Orsini E., Palazzesi C., Stella F., Prosposito P. (2012). Bragg Grating Optical Filters by UV Nanoimprinting. J. Nanomater..

[9] Royon M., Piétroy D., Marin E., Saulot A. (2017). A thermomechanical sensor using photo-inscribed volume Bragg gratings. Tribol. Int..

[10] Hill K., Meltz G. (1997). Fiber Bragg grating technology fundamentals and overview. J. Light. Technol..

[11] Sanchez C., Julián B., Belleville P., Popall M., Julián-López B. (2005). Applications of hybrid organic–inorganic nanocomposites. J. Mater. Chem..

[12] Kaczmarek H., Galka P. (2008). Effect of irgacure 651 initiator on poly(methyl methacryltate) photostability studied by UV-Vis spectroscopy. Open Process Chem. J..

[13] Segurola J., Allen N.S., Edge M., Roberts I. (1999). Photochemistry and photoinduced chemical crosslinking activity of acrylated prepolymers by several commercial type I far UV photoinitiators. Polym. Degrad. Stab..

[14] Grigorescu A.E., Hagen C.W. (2009). Resists for sub-20-nm electron beam lithography with a focus on HSQ: State of the art. Nanotechnology.

[15] Brigo L., Pistore A., Grenci G., Carpentiero A., Romanato F., Brusatin G. (2010). New hybrid organic–inorganic sol–gel positive resist. Microelectron. Eng..

[16] Brigo L., Grenci G., Carpentiero A., Pistore A., Tormen M., Guglielmi M., Brusatin G. (2011). Positive resist for UV and X-ray lithography synthesized through sol–gel chemistry. J. Sol-Gel Sci. Technol..

[17] Brusatin G., Della Giustina G., Romanato F., Guglielmi M. (2008). Design of hybrid sol–gel films for direct x-ray and electron beam nanopatterning. Nanotechnol..

[18] Innocenzi P., Malfatti L., Kidchob T., Costacurta S., Falcaro P., Marmiroli B., Cacho-Nerin F., Amenitsch H. (2011). Densification of sol–gel silica thin films induced by hard X-rays generated by synchrotron radiation. J. Synchrotron Radiat..

[19] Soppera O., Croutxé-Barghorn C. (2003). Real-time Fourier transform infrared study of the free-radical ultraviolet-induced polymerization of a hybrid sol-gel. II. The effect of physicochemical parameters on the photopolymerization kinetics. J. Polym. Sci. Part A: Polym. Chem..

[20] Girard S., Kuhnhenn J., Gusarov A., Brichard B., Van Uffelen M., Ouerdane Y., Boukenter A., Marcandella C. (2013). Radiation Effects on Silica-Based Optical Fibers: Recent Advances and Future Challenges. IEEE Trans. Nucl. Sci..

[21] Royon M., Marin E., Girard S., Boukenter A., Ouerdane Y., Stoian R. (2019). X-ray preconditioning for enhancing refractive index contrast in femtosecond laser photo-inscription of embedded waveguides in pure silica. Opt. Mater. Express.

[22] Ulrich R., Torge R. (1973). Measurement of Thin Film Parameters with a Prism Coupler. Appl. Opt..

[23] Pevere F., Von Treskow C., Marino E., Anwar M., Bruhn B., Sychugov I., Linnros J. (2018). X-ray radiation hardness and influence on blinking in Si and CdSe quantum dots. Appl. Phys. Lett..

[24] Murashkevich A.N., Lavitskaya A.S., Barannikova T.I., Zharskii I.M. (2008). Infrared absorption spectra and structure of TiO2-SiO2 composites. J. Appl. Spectrosc..

[25] Franc J., Blanc D., Zerroukhi A., Chalamet Y., Last A., Destouches N. (2006). Organo-silica–titania nanocomposite elaborated by sol–gel processing with tunable optical properties. Mater. Sci. Eng. B.

[26] Huang C., Bai H., Huang Y., Liu S., Yen S., Tseng Y. (2012). Synthesis of neutral SiO2/TiO2 hydrosol and its application as antireflective self-cleaning thin film. Int. J. Photoenergy.

